# Catatonia Spectrum: Validation of a Questionnaire Investigating Catatonia Spectrum

**DOI:** 10.3389/fpsyt.2022.913286

**Published:** 2022-05-12

**Authors:** Liliana Dell’Osso, Giulia Amatori, Andrea Cappelli, Ivan Mirko Cremone, Gabriele Massimetti, Davide Gravina, Benedetta Nardi, Francesca Benedetti, Ilaria Chiarantini, Mario Luciano, Isabella Berardelli, Natascia Brondino, Marianna De Gregorio, Giacomo Deste, Marta Nola, Antonino Reitano, Maria Rosaria Anna Muscatello, Maurizio Pompili, Pierluigi Politi, Antonio Vita, Claudia Carmassi, Mario Maj

**Affiliations:** ^1^Department of Clinical and Experimental Medicine, University of Pisa, Pisa, Italy; ^2^Department of Psychiatry, University of Campania “Luigi Vanvitelli,” Naples, Italy; ^3^Department of Neuroscience, Mental Health and Sense Organs, University of Roma “La Sapienza,” Roma, Italy; ^4^Department of Brain and Behavioral Sciences, University of Pavia, Pavia, Italy; ^5^Department of Biomedical and Dental Sciences and Morphofunctional Imaging, University of Messina, Messina, Italy; ^6^Department of Clinical and Experimental Sciences, University of Brescia, Brescia, Italy

**Keywords:** Catatonia Rating Scale, catatonia, dimensional psychiatry, validation questionnaire, psychometric instrument

## Abstract

**Aim:**

A growing body of literature has demonstrated the utility of a dimensional perspective on mental disorders. The current study aims to determine the psychometric properties of the Catatonia Spectrum (CS), a new questionnaire specifically tailored to assess the spectrum of catatonia, from full blown forms to subthreshold ones.

**Methods:**

86 adults with at least three symptom criteria for catatonia according to the Diagnostic and Statistical Manual of Mental Disorders (DSM-5), 81 adults affected by borderline personality disorder (BPD), 104 adults with a diagnosis of major depressive disorder (MDD), and 105 subjects without mental disorders (CTL), were recruited from six Italian University Departments of Psychiatry and administered the: Bush-Francis Catatonia Rating Scale (BFCRS), Bush-Francis Catatonia Screening Instrument (BFCSI), and CS.

**Results:**

CS scale demonstrated a high level of internal consistency and excellent test-retest reliability for total and domain scores. CS domain scores were positively and significantly correlated with each other (*p* < 0.001) with Pearson’s coefficients ranging from 0.337 to 0.663. All the CS domain scores were highly correlated with the CS total score. The correlation coefficients between CS and alternative measures of catatonia appeared all significant and positive. Significant differences among diagnostic groups on both CS domains and total scores were found. CS total scores increased significantly and progressively from the CTL, to the MDD and the BDP group, up to the catatonia group, which reported the highest value.

**Conclusion:**

The CS showed excellent internal consistency and test-retest reliability and strong convergent validity with alternative dimensional measures of catatonia. The questionnaire performed differently across the four diagnostic groups, with an increasing score gradient from healthy controls to patients with MDD, BPD and up to the catatonia group.

## Introduction

The word *catatonia* was first used by Kahlbaum in 1874 (1), in reference to a syndrome consisting of behavioral and motor disturbances (negativism, mutism, stereotypes, mannerisms, automatic obedience, automatisms, impulsiveness, and agitation) in combination with cognitive, affective, and neurovegetative manifestations. Differently from Kahlbaum, who ascribed to catatonia a similar nature to that of mood disorders, Kraepelin confined catatonia to one of the possible manifestations of dementia praecox (2), influencing Bleuler’s view that, in 1916, included catatonia in the group of schizophrenias (3). Kraepelin and Kahlbaum’s perspective persisted in the first four editions of the Diagnostic and Statistical Manual of Mental Diseases (DSM), and then was subjected to several criticisms (4) until it was again accorded an independent position, similar to Kahlbaum’s original view, in the fifth edition of DSM (DSM-5) (5). Currently, in the DSM-5, catatonia is included in the “Spectrum of Schizophrenia and Other Psychotic Disorders” chapter and defined by the occurrence of three or more of 12 symptoms (stupor, catalepsy, waxy flexibility, negativism, fixed posture, mannerisms, stereotypes, agitation, presence of grimacing, echolalia, and echopraxia).

Recent literature has highlighted that catatonia may be more prevalent among psychiatric patients than previously thought, particularly among younger patients (6, 7) and individuals with autism spectrum disorder (ASD) (8–12). The best pharmacological therapy of catatonia is based on the use of benzodiazepines and, in particular, Lorazepam, with 60–80% response rates (13). Furthermore, the central role of electroconvulsive therapy (ECT) in the management of catatonia is well established, with response rates of 82–96% (14) and an indication for a course of at least 5–6 applications up to a limit of 10–12 in the absence of response (15). The usefulness of ECT resides in its capacity to effectively resolve catatonic symptoms where pharmacological interventions have failed (16). In the most unfortunate cases, catatonia may develop into malignant catatonia (MC). MC represents a life-threatening condition that includes behavioral alterations, motor disturbances, and autonomic dysregulation (17). Even though the fatal consequences of MC are well established, diagnosis is frequently difficult and typically retarded (18). It is therefore understandable that the identification of a spectrum of catatonia and, in particular, the existence of an instrument designed to explore its subthreshold manifestation, may allow an early diagnosis aimed at improving patients’ treatment and prognosis. The DSM-5 describes three categories of catatonia: “catatonia associated with another mental disorder,” “catatonic disorder due to another medical condition,” and “catatonia without specification.” It is thus quite clear that the third category, comprising forms of catatonia of an unclear or insufficiently investigated nature, involves the existence of subthreshold conditions and opens the door to the definition of a spectrum that extends between the highest degrees of stupor or excitement and the subthreshold attitudes, such as staring into space or acting out of control. In this context, a self-assessment questionnaire named Catatonia Spectrum (CS) was developed to explore the symptomatology of the catatonic spectrum during the lifespan using 74 questions. The CS would therefore represent a complementary tool, and not a substitute for the diagnosis of catatonia made according to the criteria of the DSM-5, investigating, in addition to nuclear symptoms, all those subthreshold, atypical and partial manifestations that often precede the diagnosis of the disorder by years. This spectrum approach has previously been applied, successfully, to other mental disorders (19, 20).

The purpose of this study was to determine the psychometric properties of the CS.

## Materials and Methods

Data have been collected between November 2021 and January 2022 at six Italian University Departments of Psychiatry, coordinated by the University of Pisa: University of Campania “Luigi Vanvitelli,” University of Pavia, University of Messina, La Sapienza University of Rome, University of Catania, and University of Brescia.

### Study Sample and Procedures

The total sample consisted of 376 subjects distributed in four diagnostic groups, all evaluated according to DSM-5 diagnostic criteria. Exclusion criteria were: age under 18 years, language or intellectual impairment affecting the possibility to fulfill the assessments, mental disability, poor cooperation skills, and ongoing psychotic symptoms. Specifically, the four groups were individuated as follows: 86 subjects endorsing at least 3 symptom criteria for catatonia; 81 subjects diagnosed with borderline personality disorder (BPD); 104 subjects diagnosed with major depressive disorder (MDD); 105 individuals without current or lifetime mental disorders (CTL) and belonging to health care and paramedical personnel. All subjects were aged 18–60 years old and signed a written informed consent.

The Structured Clinical Interview for DSM-5, Research Version (SCID-5-RV) (21) was used to confirm the diagnoses of BPD and MDD, as well as the absence of mental disorders among CTL.

The test-retest reliability of the CS, performed in order to provide evidence for the temporal stability of the CS scores, was determined in 41 subjects randomly extracted from study sites and by means of a second evaluation over an interval of 14–21 days from the initial assessment.

The study was conducted in accordance with the Declaration of Helsinki. The Ethics Committee of the Azienda Ospedaliero-Universitaria of Pisa approved all recruitment and assessment procedures. Eligible subjects provided written informed consent, after receiving a complete description of the study and having the opportunity to ask questions. Subjects were not paid for their participation according to Italian legislation.

### Measures

Assessment procedures included the SCID-5-RD (21), the Bush-Francis Catatonia Rating Scale (BFCRS) (22), the Bush-Francis Catatonia Screening Instrument (BFCSI) (22), and the CS. Questionnaire were carried by psychiatrists who were trained and certified in the use of the study instruments.

#### The Bush-Francis Catatonia Rating Scale

The BFCRS is a rating scale developed as a tool to investigate the severity of catatonia symptoms. It consists of 23 items: (1) Immobility/Stupor; (2) Mutism; (3) Staring; (4) Posturing/Catalepsy; (5) Grimacing; (6) Echopraxia/Echolalia; (7) Stereotypy; (8) Mannerisms; (9) Stereotyped and meaningless repetition of words and phrases (Verbigeration); (10) Rigidity; (11) Negativism; (12) Waxy flexibility; (13) Withdrawal; (14) Excitement; (15) Impulsivity; (16) Automatic obedience; (17) Passive obedience; (18) Muscle resistance; (19) Motorically stuck (Ambitendency); (20) Grasp reflex; (21) Perseveration; (22) Combativeness; and (23) Autonomic abnormality. A score ranging from 0 to 3 is provided for each item. The total score is the result of the sum of the scores obtained for each of the 23 items.

#### The Bush-Francis Catatonia Screening Instrument

The BFCSI is an assessment scale developed as a screening test for the evaluation of subjects with catatonia. It consists of the first 14 items of the BFCSI. Each item (symptom) is given a score ranging from 0 (absent) to 3 (present). The presence of 2 or more of the 14 symptoms for a minimum of 24 h meets the criteria for the diagnosis of catatonia proposed by Bush et al. (22).

#### The Catatonia Spectrum

The CS is a self-assessment questionnaire that investigates nuclear, subthreshold, atypical and partial manifestations of the CS, referred to across the lifespan, divided into domains, and explored with a set of questions.

The CS consists of 74 items and is divided into 8 domains: (1) Psychomotor activity (Stupor); (2) Verbal response (Mutism); (3) Repetitive movements (Stereotypes); (4) Artificial expressions and actions (Mannerisms); (5) Oppositivity or poor response to stimuli (Negativism); (6) Response to instructions given from outside (Automatic obedience); (7) Automatisms; (8) Impulsivity. For each item there is a dichotomous answer “Yes” and “No.”

### Statistical Analyses

In order to estimate the internal consistency of the CS, the Cronbach’s alpha was calculated for each domain and for the total score of the questionnaire. The changes in alpha with deleted items were examined in order to determine how each item influenced the instrument reliability. The validity of the internal structure of the instrument was explored computing bivariate Pearson’s correlation coefficients among the eight domain scores and between each domain score and the total score. The test-retest reliability of the questionnaire was assessed by calculating the intra-class correlation coefficient (ICC) on a subgroup of 41 subjects randomly extracted from the original database and re-evaluated after an interval of 3 weeks. The convergent validity was investigated by calculating Pearson’s correlation coefficients between CS domains and total scores and BFCRS and BFCSI total score as an alternative measure of catatonic disorder. To test the discriminatory capacity of the instrument (see section “Known-Groups Validity”) the mean total and domains scores reported in the four diagnostic groups were compared through a one-way analysis of variance (ANOVA). The Bonferroni test was used for *post hoc* comparisons. All statistical analyses were performed with SPSS version 26.0 (23).

## Results

The four groups considered in the study were statistically comparable in terms of age and gender.

The catatonia group included subjects with a mean age of 40.45 years (±11.85) and consisted of 34 (39.5%) males and 52 (60.5%) females. The MDD subjects had a mean age of 40.74 (±11.46) years and consisted of 36 (34.6%) males and 68 (56.7%) females. The group of BDP subjects had a mean age of 40.54 (±13.599) years and consisted of 31 (38.3%) males and 50 (61.7%) females. The group of CTL subjects had a mean age of 37.71 (±11.02) years and consisted of 38 (36.2%) males and 67 (63.8%) females.

### Internal Consistency and Test-Retest Reliability

Table 1 shows the Cronbach’s alphas and the ICCs for the individual domains and for the total score computed on the overall sample. CS scale demonstrated a high level of internal consistency. The Cronbach’s alpha values associated with the CS domains were all good (exceeding the value of 0.7), the Cronbach’s alpha value associated with the total score of the scale was excellent (α = 0.954). Each item had a substantive correlation with the total score and provided a relevant contribution to the scale because alpha decreased when each item in turn was deleted. The test-retest reliability for total and domain scores was excellent, with all ICCs above the value of 0.90.

**TABLE 1:** Catatonia Spectrum internal consistency and test-retest reliability.

CS domains	Number of items	Cronbach’s alpha	ICC
Psychomotor activity	16	0.866	0.952
Mutism	9	0.813	0.971
Stereotypes	7	0.710	0.971
Mannerisms	7	0.785	0.968
Negativism	7	0.728	0.953
Automatic obedience	6	0.716	0.958
Automatism	10	0.811	0.942
Impulsiveness	12	0.847	0.950
Total score	74	0.954	0.984

### Validity of the Internal Structure

The CS domain scores were positively and significantly correlated with each other (*p* < 0.001) with Pearson’s coefficients ranging from 0.337 to 0.663. All the CS domain scores were highly correlated with the CS total score (see Table 2).

**TABLE 2:** Correlations among the CS domains^a^.

CS domains	Psychomotor activity	Mutism	Stereo types	Mannerisms	Negativism	Automatic obedience	Automatism	Impulsiveness
Psychomotor activity								
Mutism	0.736							
Stereotypes	0.550	0.503						
Mannerisms	0.483	0.438	0.615					
Negativism	0.607	0.575	0.507	0.465				
Automatic obedience	0.446	0.436	0.363	0.335	0.337			
Automatism	0.663	0.588	0.635	0.567	0.568	0.507		
Impulsiveness	0.611	0.511	0.531	0.576	0.623	0.337	0.612	
Total score	0.872	0.796	0.738	0.700	0.759	0.580	0.839	0.806

*^a^Pearson’s correlation coefficients were all significant at the p < 0.01 level, two-tailed.*

### Convergent Validity

Table 3 shows Pearson’s correlation coefficients for the relationships between CS total and alternative measures of catatonia. Although the correlation coefficients appear not too strong, they are all significant and positive.

**TABLE 3:** Correlations between the CS domains and BFCRS and BFCSI total score^a^.

CS domains	BFCRS total score	BFCSI total score
Psychomotor activity	0.306	0.320
Mutism	0.279	0.288
Stereotypes	0.234	0.250
Mannerisms	0.248	0.271
Negativism	0.193	0.219
Automatic obedience	0.154	0.170
Automatism	0.277	0.301
Impulsiveness	0.285	0.297
Total score	0.331	0.352

*^a^Pearson’s correlation coefficients were all significant at the p < 0.01 level, two-tailed.*

### Known-Groups Validity

Because not all of the variables analyzed had a Gaussian distribution, comparisons were performed using the non-parametric Kruskal–Wallis test, followed by *post hoc* comparisons using Dunn’s test.

Kruskal–Wallis analysis found many significant differences among diagnostic groups on both CS domain and total scores (see Table 4). Specifically, it was observed that the CS total score increases significantly and progressively, passing respectively from the CTL, to MDD, to BPD up to the catatonia group, which had the highest value.

**TABLE 4:** Comparison of CS total and domain scores among diagnostic groups.

CS domains	CTL (mean ± SD), MR^a^	Border (mean ± SD), MR	MD (mean ± SD), MR	Catatonic (mean ± SD), MR	*F*(3,372)	*p*	*Post hoc* comparison^b^
Psychomotor activity	3.64 ± 2.96, 97.36	8.42 ± 3.63, 214.57	7.92 ± 4.14, 204.72	9.99 ± 3.86, 256.62	53.70	<0.001	CTL < MDD < catatonia
Mutism	2.15 ± 1.99, 123.12	4.23 ± 2.43, 207.86	3.72 ± 2.71, 185.95	5.44 ± 2.59, 253.17	29.81	<0.001	CTL < BDP, CTL < MDD, CTL < catatonia, BPD and MDD < catatonia
Stereotypes	1.19 ± 1.42, 138.02	2.32 ± 1.78, 208.20	1.83 ± 1.79, 175.99	3.10 ± 1.99, 246.71	20.10	<0.001	CTL < BDP, CTL < catatonia, MDD < catatonia
Mannerisms	0.63 ± 1.45, 134.77	2.04 ± 1.98, 223.34	1.12 ± 1.69, 160.47	2.67 ± 2.04, 255.19	26.56	<0.001	CTL < BPD, CTL < catatonia, MDD < BPD, MDD < catatonia
Negativism	1.95 ± 1.62, 138.24	3.68 ± 2.05, 227.88	2.77 ± 2.20, 178.65	3.60 ± 1.98, 224.68	16.05	<0.001	CTL < BPD, CTL < MDD, CTL < catatonia, MDD < BPD, MDD < catatonia
Automatic obedience	2.46 ± 1.58, 160.13	3.22 ± 1.70, 204.98	2.77 ± 1.93, 177.92	3.60 ± 1.98, 220.41	6.42	<0.001	CTL < BPD, CTL < catatonia, MDD < catatonia
Automatism	2.59 ± 2.34, 129.57	4.67 ± 2.45, 212.62	3.67 ± 2.91, 170.62	6.07 ± 2.73, 259.35	30.51	<0.001	CTL < BPD, CTL < catatonia, MDD < catatonia
Impulsiveness	2.09 ± 2.36, 113.82	6.20 ± 3.28, 244.57	3.89 ± 3.31, 173.52	6.27 ± 3.43, 244.98	39.85	<0.001	CTL < BPD, CTL < MDD, CTL < catatonia, MDD < catatonia
Total score	16.69 ± 11.52	34.78 ± 13.66	27.69 ± 16.17	40.66 ± 14.26	51.76	<0.001	CTL < MDD, CTL < BPD, CTL < catatonia, MDD < BPD MDD < catatonia

*^a^MR, mean rank.*

*^b^p < 0.05.*

**FIGURE 1:**
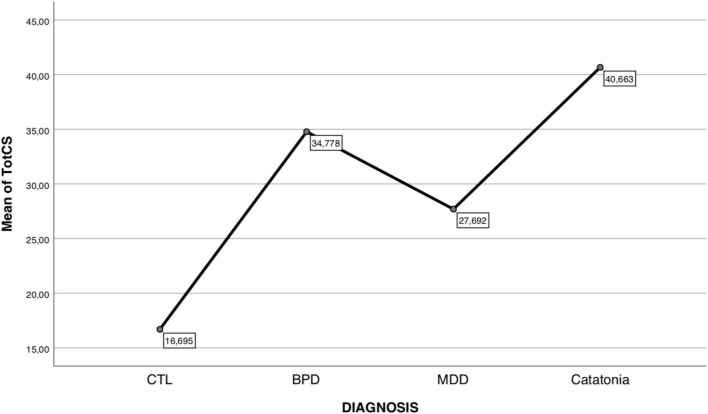
Graphic showing CS total scores observed in the diagnostic groups.

Figure 1 illustrates this trend. The domain scores, albeit with some differentiation, also follow this trend. To note that there was a non-significant difference between the BPD and catatonia groups in the scores of the Mannerism, Negativism, Automatic obedience, and Impulsiveness. It was also observed that there was no significant difference between BPD and MDD groups on the Mutism, Stereotypes, and Automatism domain scores.

## Discussion

This article introduces the CS, a new questionnaire focused on a dimensional approach to catatonia built upon a spectrum model (24–26), aiming to explore not only the core symptoms, but also the subthreshold, atypical and partial manifestations of catatonia. Results provide evidence of the validity and reliability of the CS administered to subjects endorsing at least three symptom criteria for catatonia, patients diagnosed with MDD, and BPD, as well as to healthy controls. We found excellent internal consistency and test-retest reliability and a significant and positive convergent validity with alternative dimensional measures of catatonia. As expected, the questionnaire performed differently among the four groups explored, with a progressive increase of the CS score going from healthy controls to MDD patients up to BPD subjects and catatonic ones.

The CS scores showed a positive and significant correlation with alternative dimensional measures currently adopted to assess catatonic symptoms and features. The correlation coefficients between CS and the other two measures for catatonic symptoms (BFCRS and BFCSI), although positive and significant, were not particularly strong. This could be explained by two issues: first, the CS represents a lifetime assessment instrument of catatonic symptoms, unlike the other two questionnaires that investigate catatonic manifestations at the time of assessment; second, the CS is a dimensional assessment tool that, unlike the BFCRS and BFCSI, explores the whole spectrum of catatonia, from subthreshold manifestations to the full-blown picture.

The presence of catatonic manifestations in other severe mental disorders, as found in the present study, is in agreement with data from previous literature. About 10% of patients with severe acute psychiatric illness exhibit a cluster of motor signs (mutism, negativism, rigidity, posturing, stereotypy, staring, etc.) that are identified as catatonia (27), a very life-threatening condition for the subject. Even according to the DSM-5, catatonia is typically diagnosed in hospitalized patients and most cases involve individuals with depressive or bipolar disorders (5). The availability of an instrument, capable of detecting even the most subtle manifestations of this dangerous syndrome in a patient with another mental disorder, could be fundamental to early diagnosis and prognosis.

In the present work, the highest levels of catatonic manifestations were found in patients affected by BPD, a very severe mental disorders: 80% of patients affected by BPD have suicidal behaviors or attempted suicide and 4–9% of them die by suicide (28, 29). For these reasons, the management of BPD represents one of the greatest challenges in modern psychiatry. The finding of a gradient in severity of catatonic symptoms from healthy controls to depressed subjects, with a peak reached in borderline patients, may suggest the hypothesis of a psychopathological trajectory in which the various mental disorders are placed along a continuum of severity. Therefore, the closeness of the CS score between BPD and catatonia may be due to the fact that, according to our hypothesis of a psychopathological trajectory progressing and culminating in the manifestations of the catatonic spectrum, BPD would be at a very high level of severity. However, our finding could also be derived from overestimation, by BPD patients, of their catatonic symptoms in a self-report questionnaire and this eventuality should be included among the limitations of the present study.

When examining these results, potential limitations of the study should be acknowledged. First of all, the CS is a self-report questionnaire and it may be considered less precise compared to the evaluation of the clinician. Second, our findings did not clarify whether this continuum is uni- or multidimensional because of the relatively small sample size that prevented us from conducting a factor analysis.

In the context of the above-mentioned limitations, the CS showed good psychometric properties. Altogether, the dimensions explored by the CS seem to be important for patients with catatonia, either with sub-threshold or full-blown syndrome, and demonstrated to represent a strong construct. The administration of the CS could help address some of the weaknesses of the categorical definition of catatonia according to the DSM-5 and the tools already available, providing a more accurate description of the patient-specific clinical phenotype, with relevant implications for research and, ultimately, clinical awareness.

## Conclusion

The results of the present study provide a coherent construct of the CS with high internal consistency, sound test-retest reliability, significative and positive convergent validity with alternative dimensional measures of catatonia. Further studies will be required to test this new instrument in other diagnostic groups and in the general population.

## Data Availability Statement

The raw data supporting the conclusions of this article will be made available by the authors, without undue reservation.

## Ethics Statement

The studies involving human participants were reviewed and approved by Comitato Etico Regionale per la Sperimentazione Clinica della Regione Toscana. The patients/participants provided their written informed consent to participate in this study.

## Author Contributions

LD’O conceived the work. All authors collected the data processed in the study. GM did statistical analysis. GA, AC, and LD’O drafted the manuscript. CC and LD’O revised the work. All authors provided approval of the version to be published.

## Conflict of Interest

The authors declare that the research was conducted in the absence of any commercial or financial relationships that could be construed as a potential conflict of interest.

## Publisher’s Note

All claims expressed in this article are solely those of the authors and do not necessarily represent those of their affiliated organizations, or those of the publisher, the editors and the reviewers. Any product that may be evaluated in this article, or claim that may be made by its manufacturer, is not guaranteed or endorsed by the publisher.
