# Supplementary material for: Catatonia Spectrum: Validation of a Questionnaire Investigating Catatonia Spectrum
Source: Front Psychiatry. 2022 May 12;13:913286. doi: 10.3389/fpsyt.2022.913286 (PMC9133529; doi:10.3389/fpsyt.2022.913286)
Supplement: Supplementary file 1 [file Presentation_1.pdf]

1. Appendix Subject

CATATONIA SPECTRUM (CS)

Subject ID:________________

Date:________________

Instructions: the following questions refer to feelings or experiences you may have had in the past or that you may be experiencing currently. Please answer each of the questions by circling “Yes” or “No”.

DOMAIN I: PSYCHOMOTOR ACTIVITY

Stupor

Have you ever noticed or have your friends/relatives ever told you that you…

1	Stare into space or stare at the sky?	Yes	No
2	Feel slowed down or have difficulty in doing daily activities such as brushing your teeth, shaving, wearing make-up, combing your hair or choosing clothes?	Yes	No
3	Feel inhibited in making choices, even the simple ones?	Yes	No
4	Feel you would be in trouble if you didn’t have a list of the things to do every day, such as buying groceries, going to the doctor, meeting someone?	Yes	No

5	Move slowly in the daily activities, such as eating, speaking, reading, writing, dressing or taking a bath?	Yes	No
6	Have a fixation to ruminate with certain topics?	Yes	No
7	Feel tired, exhausted or weak, as if every small task, such as washing your face or setting the table, were a great effort?	Yes	No
8	Have no emotions as before?	Yes	No
9	Get the impression that the world around was unreal or weird, like there was a veil between you and the surrounding environment or that you were in a dream?	Yes	No
10	Get the impression that the objects around were smaller and farther away?	Yes	No
11	Get the impression that your body or parts of it were unreal, inanimate or not organic (e.g. wooden)?	Yes	No
12	Get the impression that you didn't feel any emotion when you were crying or laughing?	Yes	No
13	Have difficulty sleeping because you were unable to stop thinking?	Yes	No
14	Have difficulty or need help bathing?	Yes	No
15	Feel lazy, unable to comply with commitments?	Yes	No
16	Be immobilized, unable to respond to calls or do what was asked of you?	Yes	No

DOMAIN II: VERBAL RESPONSE

Mutism

Have you ever noticed or have your friends/relatives ever told you that you…

17	Are taciturn and, sometimes, unable to speak?	Yes	No
18	Use very short sentences?	Yes	No
19	Speak in a low or weird monotonous voice?	Yes	No
20	Make faces and/or strange gestures?	Yes	No
21	Have difficulty thinking what to say during a conversation?	Yes	No
22	Have difficulty putting your feelings into words?	Yes	No
23	Have no thought, so much so that during a conversation, you have the impression that the words were spoken by a robot?	Yes	No
24	Are silent for a long time or had been invited by others to break a long silence?	Yes	No
25	Are unable to communicate verbally for a shorter or longer period of time?	Yes	No

DOMAIN III: REPETITIVE MOVEMENTS

Stereotypes

Have you ever noticed or have your friends/relatives ever told you that you…

26	Repeat an action several times (e.g. wringing hands, turning an object or twisting a lock of hair between your fingers, squinting) for no specific reason?	Yes	No
27	Walk in a particular way?	Yes	No
28	Repeatedly touch an object or part of your body for no apparent reason?	Yes	No
29	Clear your nose or your throat before starting to speak, make particular hand movements before writing or knocking on the door?	Yes	No
30	Have a tendency to fiddle with the food on your plate?	Yes	No
31	Go over the letters or words already written several times with the pen?	Yes	No
32	Repeat an unchanged and constant sequence of one or more movements for no apparent reason?	Yes	No

DOMAIN IV : ARTIFICIAL EXPRESSIONS AND ACTIONS

Mannerisms

Have you ever noticed or have your friends/relatives ever told you that you…

33	Behave in a manner that was considered too ceremonious and / or speak in a formal and obsequious way?	Yes	No
34	Amplify some daily gestures, making them almost caricatured?	Yes	No
35	Behave with others in a theatrical way, accentuating the movements or overloading the pronunciation of some words?	Yes	No
36	Greet others with broad smiles or by making more or less deep bows?	Yes	No
37	Make inappropriate statements or engage in bizarre behavior?	Yes	No
38	Anticipate / express with gestures what you wanted to express verbally?	Yes	No
39	Perform strange and exaggerated actions?	Yes	No

DOMAIN V: OPPOSITIVENESS OR POOR STIMULUS - RESPONSE

Negativism

Have you ever noticed or have your friends/relatives ever told you that you…

40	Feel discomfort in front of physical manifestations of affection (caresses, hugs, kisses) from relatives or friends?	Yes	No
41	Refuse, without particular reasons, to satisfy the requests of others?	Yes	No
42	Tend to disagree or act like a ”Contrary Mary?”	Yes	No
43	Prefer solitary activities and / or be comfortable alone?	Yes	No
44	React aggressively when forced to do something?	Yes	No
45	Involuntarily resist a physical movement such as a push or a hug or another type of movement induced by others?	Yes	No
46	Take opposing attitudes to those suggested from the outside or totally refuse to answer?	Yes	No

DOMAIN VI: RESPONSE TO INSTRUCTIONS GIVEN FROM OUTSIDE

Automatic obedience

Have you ever noticed or have your friends/relatives ever told you that you…

47	Get involved in things you didn't want to do because you couldn't say no?	Yes	No
48	Feel uncomfortable in expressing or avoid expressing your disagreement or disapproval of others?	Yes	No
49	Apologize even when not necessary?	Yes	No
50	Obey authority without objecting?	Yes	No
51	Feel pushed to do something as if you were being moved by an external force?	Yes	No
52	Obey unconditionally the instructions that were suggested to you from the outside?	Yes	No

DOMAIN VII: AUTOMATISMS

Have you ever noticed or have your friends/relatives ever told you that you…

53	Carry out a series of fixed and pre-established operations in different situations and circumstances?	Yes	No
54	Have difficulty making changes to your daily habits?	Yes	No
55	Have difficulty in changing your point of view, even when you were wrong?	Yes	No
56	Have difficulty in changing your way of acting and / or your methods, even when there could have been a better system?	Yes	No
57	Stay in the same position during sleep?	Yes	No
58	Feel like you were doing things automatically without thinking about it?	Yes	No
59	Repeat a slogan or a musical tune, as if you couldnt get it off your mind?	Yes	No
60	Be pushed or feel inclined to imitate the words / phrases just spoken by others?	Yes	No
61	Be pushed or feeling the propensity to repeat or imitate the gestures / movements just made by others?	Yes	No
62	Feel the need or find yourself taking actions automatically, even without a specific purpose?	Yes	No

DOMAIN VIII: IMPULSIVITY

Have you ever noticed or have your friends/relatives ever told you that you…

63	Have outbursts of anger, for no real reason?	Yes	No
64	Fail to be patient?	Yes	No
65	Have the impression that your emotions were not under control?	Yes	No
66	Feeling extremely excited, out of control?	Yes	No
67	Feel the need to drink alcohol to control your agitation?	Yes	No
68	Do something sudden that others didn't expect?	Yes	No
69	Swear or say bad words, behaving in a completely unusual way for you?	Yes	No
70	Have impulsive behaviors that others considered inappropriate or repent after carrying out actions considered impulsive?	Yes	No
71	Become violent, aggressive or lose control, even without reason?	Yes	No
72	Suddenly feel the urge to run, scream or take off your clothes?	Yes	No

73	Have the impulse to harm yourself (e.g. scratching, cutting, burning) and / or others, in moments of poor self-control?	Yes	No
74	Feeling extremely agitated, as if it were not in itself?	Yes	No
